# Data correlations between gender, cytomegalovirus infection and T cells, NK cells, and soluble immune mediators in elderly humans

**DOI:** 10.1016/j.dib.2016.06.006

**Published:** 2016-06-15

**Authors:** Ahmad Al-Attar, Steven R. Presnell, Charlotte A. Peterson, D. Travis Thomas, Charles T. Lutz

**Affiliations:** a Department of Pathology and Laboratory Medicine, College of Medicine, University of Kentucky, Lexington, KY, USA; b Department of Rehabilitation Sciences, College of Health Sciences, University of Kentucky, Lexington, KY, USA; c Department of Clinical Sciences, College of Health Sciences, University of Kentucky, Lexington, KY, USA; d Department of Microbiology, Immunology and Molecular Genetics, College of Medicine, University of Kentucky, Lexington, KY, USA

**Keywords:** NK cell, Ageing, Cytomegalovirus, T cell, Sex factors, Vitamin D, C-reactive protein, Adiponectin, IL-15, Sphingosine-1-phosphate, Retinol binding protein-4

## Abstract

We describe a cohort of 50 elderly subjects, age at least 70 years. We present gender-specific findings in T lymphocyte markers and soluble immune mediators. We show the correlation between cytomegalovirus infection status with CD56^dim^ NK cell responses to a variety of stimuli and with CD56^bright^/CD56^dim^ NK cell ratio. We also present the correlation of retinol binding protein (RBP)−4 plasma levels with NK cell responses and we explore the relationship between gender and adiponectin, 25(OH)D (vitamin D), and RBP4 in affecting CD56^dim^ NK cell responses. These data are discussed in Al-Attar et al. (2016) [1].

**Specifications Table**

**Table:** 

Subject area	*Immunology*
More specific subject area	*Sex differences in immune cells and soluble mediators*
Type of data	*Tables*
How data was acquired	*Flow cytometry and enzyme-linked immunosorbent assay*
Data format	*Analyzed*
Experimental factors	*Immune cells were analyzed immediately ex vivo or were stimulated in vitro*
Experimental features	*Correlations and mean differences were calculated*
Data source location	*Lexington, KY – USA*
Data accessibility	*Data are within this article*

**Value of the data**

• Researchers need to be aware of the gender differences in NK cell responses to various stimuli.
• Exposure to cytomegalovirus (CMV) affects immune responses from T and NK cells and could therefore be an important factor to consider when performing research in elderly human subjects.
• Levels of soluble plasma immune mediators adiponectin and vitamin D (25(OH)D) which affect NK cell development and activity are higher in elderly women compared to men, and are important factors to consider when studying human NK cells**.**

## 1 Data

Enclosed are data concerning T cell markers and subsets found in elderly women and men (Table 1). Also shown is the effect of cytomegalovirus (CMV) infection on CD56^dim^ NK cell responses to a variety of stimuli and on the CD56^bright^/CD56^dim^ NK cell ratio in blood lymphocytes (Table 2). We present the levels of various plasma immune mediators and their levels in elderly women and men (Table 3). We show how plasma RBP4 level correlates with NK cell responses in vitro (Table 4) and we explore if the interaction between gender and plasma adiponectin,RBP4, and 25(OH)D (vitamin D) affects CD56^dim^ NK cell responses in vitro (Table 5). Full details of the data can be found in Al-Attar et al. [1].

## 2 Experimental design, materials and methods

Male and female subjects >70 years were recruited from volunteer donor pools at the University of Kentucky Sanders-Brown Center on Aging and by advertisements. Venous blood from 26 males (age mean±standard error of the mean (SEM), 77.8±0.31, range 70–90 years) and 24 females (age mean±SEM, 77.0±0.91, range 70–85 years) were analyzed between October 2012 and April 2014. Prospective donors were screened by telephone interview to exclude those with conditions previously demonstrated to affect NK cells. Exclusion criteria included hospitalization or serious illness in the prior year, history of immunologic illness (rheumatoid arthritis, systemic lupus, scleroderma, polymyositis, Sjögren׳s syndrome, transplantation, etc), current use of immunomodulatory medications (e.g., corticosteroids), inability to walk one city block, regular consumption of two or more alcoholic beverages per day (28 g ethanol), diabetes, and a history of cancer within the last 10 years, except non-melanoma skin cancers. Two female subjects (but no male subjects) received hormone replacement therapy, one with topical estrogen and one with topical estrogen, progesterone, and testosterone. The gender differences affecting CD56^dim^ NK cell CD38 density and the CD56^bright^ to CD56^dim^ NK cell ratio were no longer significant when the hormone replacement subjects were excluded from analysis. All other gender differences reported below remained significant after exclusion of these two subjects. Blood samples were obtained from people without acute illness in the preceding week between 9:30 a.m. and 12:45 p.m. Lymphocytes and serum 25-hydroxyvitamin D (25(OH)D) were analyzed immediately; plasma was aliquoted and stored at −80 °C. All human subjects research was approved by the University of Kentucky Institutional Review Board.

### 2.1 ex vivo staining

Whole blood was diluted 1:1 with PBS and overlaid on Lymphoprep^®^ lymphocyte separation medium (Axis-Shield, Oslo, Norway) according to the manufacturer׳s protocol. Peripheral blood mononuclear cells (PBMC) were collected and washed twice. For ex vivo staining, ~0.5×10^6^ PBMC were washed and incubated with human IgG for 15 min at room temperature to block Fc-receptor binding and then stained on ice for 30 min with combinations of fluorescently labeled mAb (See Supplementary Table S1 in Ref. [1]). After washing, the cells were analyzed on a LSR-II flow cytometer (BD, Franklin Lakes, NJ). CD4 and CD8 T cells were divided into four subpopulations, T_N_: CD62L^+^CD45RA^+^, T_CM_: CD62L^+^CD45RA^—^, T_EM_: CD62L^—^CD45RA^—^, and T_EMRA_: CD62L^—^CD45RA^+^. CD56^dim^ NK cells that did not stain positive for CD158b, CD158f, CD158e/k or NKG2A were considered unlicensed.

### 2.2 NK cell stimulation

Peripheral blood mononuclear cells (PBMC, 0.5×10^6^) were incubated in 6-well plates in media (RPMI 1640 media, 10% FBS, 20 mM glutamine, non-essential amino acids, and antibiotics) in the presence of either no stimulation, IL-2 (200,000 U/L, Biological Resources Branch, National Cancer Institute, Frederick, MD), IL-15 (100 μg/L, BioLegend), IL-12 (10 μg/L, Peprotech, Rocky Hill, NJ) plus IL-18 (100 μg/L, R&D Systems, Minneapolis, MN), overnight, or with 1×10^6^ K562 cells (E:T ratio 1:2) for 3 h at 37 °C. The final three hours of incubation were in the presence of 5 mg/L brefeldin A and 2 μM monensin (BioLegend). Cells were then washed, and stained with CD3, CD16, CD56 and CD107a (as described above), fixed in 2% paraformaldehyde solution, then permeabilized (1x Permeabilization buffer, eBioscience) and stained with anti-IFN-γ and anti-MIP-1β mAb. For anti-NKp46 stimulation, wells in 24-well plates were coated by incubation with 0.5 mL 2.5 mg/L anti-NKp46 mAb (eBioscience) in PBS overnight at 4 °C. Unbound mAb was removed by washing with PBS. Cells were cultured overnight at 37 °C in 5% CO_2_ with 500 ng/L IL-12, transferred to anti-NKp46-coated wells, cultured for 3 h, harvested, and stained as above.

### 2.3 Cytokines, soluble immune mediators, and antibodies

Following manufacturer instructions, enzyme-linked immunosorbent assay was used to measure plasma C-reactive protein (CRP) and adiponectin levels (eBioscience) and retinol-binding protein 4 (RBP4) levels (Abcam, Cambridge, England). Plasma IL-15 was quantified with the QuantiGlo Chemiluminescent Immunoassay kit (R&D Systems). Reported values are the average of a single measurement of each serum sample tested in two (IL-15), three (Adiponectin, CRP), or four (RBP4) independent experiments. For sphingosine-1-phosphate (S1P) and dihydrosphingosine-1-phosphate (dhS1P) measurements, plasma samples with added deuterated standards [2] were extracted using acidified organic solvents [3]. S1P and dhS1P 1evels were measured using high pressure liquid chromatography–electrospray ionization tandem mass spectrometry, and quantified by comparing levels to the internal standard [4]. The University of Kentucky Clinical Chemistry Laboratory measured serum 25(OH)D by liquid chromatography-tandem mass spectrometry using atmospheric pressure chemical ionization in positive ion mode and quantitated by comparison to a deuterated internal standard. Plasma samples were diluted 100-fold and anti-cytomegalovirus (CMV) IgG was measured in duplicate via binding to immobilized CMV antigen. Bound CMV IgG was detected by horseradish peroxidase conjugated anti-human IgG antibody (DRG CMV IgG ELISA, Springfield, NJ). None of the positive or negative anti-CMV IgG levels were close to the intermediate range.

### 2.4 Statistical methods

For means comparisons, data were first analyzed with Levene׳s test for equality of variance using SPSS, version 22 (IBM, Armonk, NY). When gender based variances were significantly unequal, means were compared by the nonparametric Mann–Whitney U test. None of the means analyzed by the Mann–Whitney test significantly differed by gender. Otherwise, means were compared using the 2-tailed student׳s t test. Levels are displayed as means±SEM.

## Figures and Tables

**Table 1:** Gender and T cell markers.

*Cell*	*Subset*	*Sex*	*Mean*	*SEM*	*P value*
T cell	CD4/8 ratio	♀	3.37	.45	.79
		♂	3.56	.56	
					
T cell	Ki67	♀	3.59	.22	.59
		♂	3.43	.20	
					
T cell	CD4	♀	66.67	2.68	.83
		♂	67.50	2.74	
					
CD4	CD57	♀	6.34	1.15	.51
		♂	5.35	.95	
					
CD4	CD28	♀	95.36	1.31	.46
		♂	96.55	.96	
					
CD4	HLA-DR	♀	25.74	2.05	.70
		♂	24.96	1.45	
					
CD4	HLA-DR gMFI	♀	126.98	23.02	.34
		♂	83.87	5.21	
					
CD4	CD38	♀	45.77	3.99	.12
		♂	43.52	1.44	
					
CD4	CD38 gMFI	♀	175.60	21.15	.30
		♂	138.72	5.52	
					
T cell	CD8	♀	25.96	2.36	.73
		♂	27.23	2.66	
					
CD8	CD57	♀	47.83	4.28	.92
		♂	47.20	4.51	
					
CD8	CD28	♀	50.55	5.26	.94
		♂	50.02	5.02	
					
CD8	HLA-DR	♀	65.45	3.71	.79
		♂	66.72	2.89	
					
CD8	HLA-DR gMFI	♀	294.02	35.63	.68
		♂	275.66	28.01	
					
CD8	CD38	♀	22.02	2.68	.35
		♂	17.12	1.29	
					
CD8	CD38 gMFI	♀	76.29	6.20	.27
		♂	68.71	3.65	

For each tested analyte on gated T cell subset, the mean and standard error of the mean (SEM) is shown for each gender. All values are given as percentage of the gated population, except when geometric mean fluorescence intensity (gMFI) antigen level is given. Statistical significance of mean differences was determined by student׳s T test.

**Table 2:** CMV effect on CD56^dim^ NK cell responses and on CD56^bright^/CD56^dim^ ratio.

*Analyte*	*Stimulus*	*CMV*	*Mean*	*SEM*	*Sig.*
CD107a	Nil	−	3.91	0.52	.808
		+	4.12	0.50	
	K562	−	15.89	1.80	.937
		+	15.72	1.15	
	NKp46	−	20.50	3.43	.755
		+	21.84	2.30	

IFN-γ	Nil	−	1.31	0.24	.928
		+	1.34	0.20	
	K562	−	3.46	0.64	.070
		+	2.32	0.30	
	NKp46	−	9.33	1.47	.920
		+	9.11	1.23	
	IL-2	−	2.00	0.37	.814
		+	2.12	0.28	
	IL-15	−	2.63	0.48	.342
		+	3.94	0.83	
	IL-12/18	−	20.55	4.67	.399
		+	16.54	2.32	

**MIP-1**β	**Nil**	–	**6.99**	**0.86**	**.048**
		+	**5.06**	**0.49**	
	K562	–	32.98	3.28	.599
		+	30.93	2.05	
	NKp46	−	48.92	5.82	.725
		+	46.39	3.84	
	IL-2	−	40.14	3.71	.123
		+	33.78	2.08	
	IL-15	−	60.90	4.11	.450
		+	56.99	2.76	
	IL-12/18	−	31.40	5.28	.420
		+	27.06	2.63	

Ratio*	Nil	–	.0476	.0073	.960
		+	.0471	.0046	

CD56^dim^ NK cells were gated and tested for the indicated analyte in response to the designated stimulus. Shown is the mean and standard error of the mean (SEM) for subjects who were CMV infected (+) or not CMV infected (−). Bold print denotes significant mean differences as determined by student׳s T test.

* CD56^bright^/CD56^dim^ ratio, tested immediately ex vivo.

**Table 3:** Gender and soluble immune mediators.

*Analyte*	*Sex*	*Mean*	*SEM*	*P value*
CRP	♀	2.824	0.463	.173
	♂	1.973	0.408	
				
IL-15	♀	4.718	0.210	.462
	♂	4.527	0.152	
				
**Adiponectin**	♀	**21.47**	**2.07**	**.019**
	♂	**14.13**	**2.19**	
				
S1P	♀	754.2	29.32	.642
	♂	732.2	36.3	
				
dhS1P	♀	65.30	2.90	.536
	♂	62.70	3.00	
				
S1P+dhS1P	♀	819.5	31.21	.625
	♂	794.9	38.4	
				
**RBP4**	♀	**45.80**	**1.06**	**.039**
	♂	**49.02**	**1.08**	
				
**25(OH)D**	♀	**38.35**	**2.78**	**.025**
	♂	**30.21**	**2.16**	
				
**Vitamin D Supplement**	♀	**.625**	**.101**	**.002**
	♂	**.192**	**.079**	

For each tested analyte, the mean and standard error of the mean (SEM) is shown for each gender. Analytes and their units are C-reactive protein (CRP, mg/L), IL-15 (mg/L), adiponectin (mg/L), sphingosine-1-phosphate (S1P, nM), dihydroS1P (dhS1P, nM), the sum of S1P and dhS1P (nM), RBP4 (mg/L), and vitamin D (25(OH)D, μg/L). Bold print denotes significant mean differences as determined by student׳s T test.

**Table 4:** Correlation of RBP4 levels with NK cell responses.

*NK Cell*	*Stimulus*	*Analyte*	*ρ*	*Sig.*
CD56^bright^	None	CD107a	.025	.864
CD56^bright^	K562	CD107a	.249	.082
**CD56**^**bright**^	**NKp46**	**CD107a**	−**.303**	**.033**
CD56^dim^	None	CD107a	.018	.901
CD56^dim^	K562	CD107a	.065	.656
CD56^dim^	NKp46	CD107a	−.221	.123
CD56^bright^	None	IFN-γ	−.065	.655
CD56^bright^	K562	IFN-γ	.259	.069
**CD56**^**bright**^	**NKp46**	**IFN-**γ	−**.334**	**.018**
CD56^bright^	IL-2	IFN-γ	−.223	.120
CD56^bright^	IL-15	IFN-γ	−.180	.212
CD56^bright^	IL-12/18	IFN-γ	−.228	.111
CD56^dim^	None	IFN-γ	.038	.795
CD56^dim^	K562	IFN-γ	.142	.325
**CD56**^**dim**^	**NKp46**	**IFN-**γ	−**.308**	**.030**
CD56^dim^	IL-2	IFN-γ	−.022	.879
CD56^dim^	IL-15	IFN-γ	−.194	.177
CD56^dim^	IL-12/18	IFN-γ	−.234	.101
CD56^bright^	None	MIP-1β	−.177	.219
CD56^bright^	K562	MIP-1β	.253	.076
CD56^bright^	NKp46	MIP-1β	−.246	.086
**CD56**^**bright**^	**IL-2**	**MIP-1**β	**.336**	**.017**
CD56^bright^	IL-15	MIP-1β	.238	.096
CD56^bright^	IL-12/18	MIP-1β	−.131	.363
CD56^dim^	None	MIP-1β	−.262	.066
CD56^dim^	K562	MIP-1β	−.089	.537
**CD56**^**dim**^	**NKp46**	**MIP-1**β	−**.300**	**.034**
CD56^dim^	IL-2	MIP-1β	−.012	.933
CD56^dim^	IL-15	MIP-1β	−.127	.379
**CD56**^**dim**^	**IL-12/18**	**MIP-1**β	−**.284**	**.046**

Shown are nonparametric Spearman׳s correlation coefficients (*ρ*) and significance (Sig.) between RBP4 level and the indicated parameter. Bold print denotes significant correlations.

**Table 5:** The influence of Adiponectin, RBP4, and 25(OH)D levels on NK cell responses does not outweigh sex effect.

Model		Unstandardized coefficients		Standardized coefficients	t	Sig.
		B	Std. error	Beta		
**Dependent Variable: % CD107a on K562-stimulated CD56**^**dim**^**NK cells**						
1	(Constant)	17.971	1.324		13.574	.000
	**Sex**	−4.232	1.836	−.316	−2.305	**.026**
2	(Constant)	18.462	2.324		7.944	.000
	**Sex**	−4.400	1.965	−.328	−2.240	**.030**
	Adiponectin	−.023	.089	−.038	−.258	.797

**Dependent Variable: % CD107a on K562-stimulated CD56**^**dim**^**NK cells**						
1	(Constant)	17.971	1.324		13.574	.000
	**Sex**	−4.232	1.836	−.316	−2.305	**.026**
2	(Constant)	9.696	8.085		1.199	.236
	**Sex**	−4.814	1.918	−.359	−2.510	**.016**
	RBP4	.181	.174	.148	1.037	.305

**Dependent Variable: % CD107a on K562-stimulated CD56**^**dim**^**NK cells**						
1	(Constant)	17.417	1.329		13.106	.000
	**Sex**	−3.942	1.860	−.301	−2.120	**.040**
2	(Constant)	19.254	3.337		5.770	.000
	**Sex**	−4.332	1.982	−.331	−2.185	**.034**
	25(OH)D	−.048	.080	−.091	−.601	.551

**Dependent Variable: % IFN-**γ **positive on NKp46-stimulated CD56**^**dim**^**NK cells**						
1	(Constant)	11.182	1.354		8.255	.000
	**Sex**	−3.870	1.878	−.285	−2.060	**.045**
2	(Constant)	9.594	2.362		4.061	.000
	Sex	−3.327	1.997	−.245	−1.666	.102
	Adiponectin	.074	.090	.121	.822	.415

**Dependent Variable: % IFN-**γ **positive on NKp46-stimulated CD56**^**dim**^**NK cells**						
1	(Constant)	11.182	1.354		8.255	.000
	**Sex**	−3.870	1.878	−.285	−2.060	**.045**
2	(Constant)	22.885	8.185		2.796	.007
	Sex	−3.047	1.942	−.224	−1.569	.123
	RBP4	−.256	.176	−.207	−1.449	.154

**Dependent Variable: % IFN-**γ **positive on NKp46-stimulated CD56**^**dim**^**NK cells**						
1	(Constant)	11.433	1.409		8.112	.000
	Sex	−3.862	1.972	−.280	−1.958	.056
2	(Constant)	9.147	3.534		2.589	.013
	Sex	−3.377	2.099	−.245	−1.609	.115
	25(OH)D	.060	.084	.108	.706	.484

**Dependent Variable: % MIP-1**β **positive on unstimulated CD56**^**dim**^**NK cells**						
1	(Constant)	6.849	.591		11.591	.000
	**Sex**	−2.395	.819	−.389	−2.923	**.005**
2	(Constant)	6.766	1.038		6.520	.000
	**Sex**	−2.367	.877	−.384	−2.697	**.010**
	Adiponectin	.004	.040	.014	.097	.923

**Dependent Variable: % MIP-1**β **positive on unstimulated CD56**^**dim**^**NK cells**						
1	(Constant)	6.849	.591		11.591	.000
	**Sex**	−2.395	.819	−.389	−2.923	**.005**
2	(Constant)	10.902	3.600		3.028	.004
	**Se**x	−2.110	.854	−.342	−2.470	**.017**
	RBP4	−.089	.078	−.158	−1.141	.260

**Dependent Variable: % MIP-1**β **positive on unstimulated CD56**^**dim**^**NK cells**						
1	(Constant)	6.877	.615		11.186	.000
	**Sex**	−2.277	.860	−.367	−2.647	**.011**
2	(Constant)	5.671	1.537		3.689	.001
	**Sex**	−2.021	.913	−.326	−2.213	**.032**
	25(OH)D	.031	.037	.126	.857	.396

**Dependent Variable: % MIP-1**β **positive on K562-stimulated CD56**^**dim**^**NK cells**						
1	(Constant)	35.617	2.380		14.965	.000
	**Sex**	−7.917	3.300	−.327	−2.399	**.020**
2	(Constant)	32.893	4.152		7.922	.000
	Sex	−6.985	3.510	−.289	−1.990	.052
	Adiponectin	.127	.158	.116	.802	.427

**Dependent Variable: % MIP-1**β **positive on K562-stimulated CD56**^**dim**^**NK cells**						
1	(Constant)	35.617	2.380		14.965	.000
	**Sex**	−7.917	3.300	−.327	−2.399	**.020**
2	(Constant)	30.912	14.684		2.105	.041
	**Sex**	−8.247	3.484	−.341	−2.367	**.022**
	RBP4	.103	.316	.047	.325	.747

**Dependent Variable: % MIP-1**β **positive on K562-stimulated CD56**^**dim**^**NK cells**						
1	(Constant)	34.600	2.336		14.812	.000
	**Sex**	−7.871	3.269	−.338	−2.408	**.020**
2	(Constant)	31.242	5.863		5.328	.000
	**Sex**	−7.158	3.483	−.307	−2.055	**.046**
	25(OH)D	.088	.140	.093	.625	.535

**Dependent Variable: % MIP-1**β **positive on NKp46-stimulated CD56**^**dim**^**NK cells**						
1	(Constant)	54.117	4.426		12.226	.000
	**Sex**	−13.494	6.138	−.302	−2.198	**.033**
2	(Constant)	46.187	7.645		6.041	.000
	Sex	−10.781	6.464	−.242	−1.668	.102
	Adiponectin	.369	.291	.184	1.268	.211

**Dependent Variable: % MIP-1**β **positive on NKp46-stimulated CD56**^**dim**^**NK cells**						
1	(Constant)	54.117	4.426		12.226	.000
	**Sex**	−13.494	6.138	−.302	−2.198	**.033**
2	(Constant)	105.250	26.274		4.006	.000
	Sex	−9.899	6.233	−.222	−1.588	.119
	RBP4	−1.117	.566	−.276	−1.973	.054

**Dependent Variable: % MIP-1**β **positive on NKp46-stimulated CD56**^**dim**^**NK cells**						
1	(Constant)	52.922	4.529		11.686	.000
	Sex	−11.005	6.337	−.251	−1.737	.089
2	(Constant)	48.312	11.393		4.241	.000
	Sex	−10.027	6.768	−.228	−1.482	.146
	25(OH)D	.120	.272	.068	.442	.661

**Dependent Variable: % MIP-1**β **positive on IL-15-stimulated CD56**^**dim**^**NK cells**						
1	(Constant)	63.646	3.149		20.213	.000
	**Sex**	−10.688	4.367	−.333	−2.448	**.018**
2	(Constant)	62.546	5.528		11.315	.000
	**Sex**	−10.312	4.673	−.321	−2.207	**.032**
	Adiponectin	.051	.211	.035	.243	.809

**Dependent Variable: % MIP-1**β **positive on IL-15-stimulated CD56**^**dim**^**NK cells**						
1	(Constant)	63.646	3.149		20.213	.000
	**Sex**	−10.688	4.367	−.333	−2.448	**.018**
2	(Constant)	58.325	19.434		3.001	.004
	**Sex**	−11.062	4.611	−.345	−2.399	**.020**
	RBP4	.116	.419	.040	.278	.783

**Dependent Variable: % MIP-1**β **positive on IL-15-stimulated CD56**^**dim**^**NK cells**						
1	(Constant)	63.191	3.173		19.917	.000
	Sex	−8.766	4.440	−.282	−1.974	.054
2	(Constant)	62.584	7.998		7.825	.000
	Sex	−8.637	4.751	−.278	−1.818	.076
	25(OH)D	.016	.191	.013	.083	.934

Multivariate analysis of sex-specific NK cell responses. Each dependent variable (NK cell response) was compared with the independent variable, sex (Model 1, Pearson correlation), or with sex along with other independent variables, either adiponectin, RBP4, or 25(OH)D (vitamin D) plasma levels (Model 2, multiple linear regression). For scoring purposes, males=1; females=0. Therefore, a negative correlation with sex indicates stronger responses in women than in men. 25(OH)D levels were not available for 1 female and 2 male subjects. Therefore, the significance (Sig.) of the correlation between sex and the NK cell response is different than when adiponectin and RBP4 effects are analyzed. The value of the unstandardized coefficient (B) reflects the amount of change in the predicted preference ranking. Using the standardized coefficient (Beta), interpretations are based on the standard deviation (SD) of the variable. Each standardized coefficient (Beta) indicates the number of SD that the dependent variable changes for a 1 SD change in the independent variable, the other independent variable remaining constant. For both B and Beta, the higher the absolute value (positive or negative), the greater the effect of the given independent variable on the dependent variable. For example, in the first table (Model 2), the absolute value of B and Beta for Sex was significantly greater than B and Beta for Adiponectin, indicating that Sex had a stronger (inverse) correlation with the NK cell response than did Adiponectin. Significant values (as determined by Pearson correlation, Model 1, and multiple linear regression, Model 2) are shown in bold print.
