# Supplementary material for: Data correlations between gender, cytomegalovirus infection and T cells, NK cells, and soluble immune mediators in elderly humans
Source: Data Brief. 2016 Jun 15;8:536–44. doi: 10.1016/j.dib.2016.06.006 (PMC4961800; doi:10.1016/j.dib.2016.06.006)
Supplement: Supplementary file 1 — Supplementary material [file mmc1.docx]

*Data article*

**Title: *Data Correlations between Gender, Cytomegalovirus Infection and T cells, NK cells, and Soluble Immune Mediators in Elderly Humans***

**Authors:** Ahmad Al-Attar ^a^, Steven R. Presnell ^a^, Charlotte A. Peterson ^b^, D. Travis Thomas ^c^, Charles T. Lutz ^a,d,^*

**Contact email:** charles.lutz@uky.edu

Conflicts of interest: none
